# 

**Published:** 1874-02

**Authors:** 


### MISSISSIPPI VALLEY DENTAL SOCIETY.

The regular annual meeting of this Society will be held in the city of St. Louis, Mo., on the first Wednesday of March next, at 10 o'clock A. M., and will continue two or three days. It meets in connection with the Missouri State Dental Society, and a grand good time is expected. This is a new move for the old Miss. V. Society ; its meetings have, from its organization, been held in Cincinnati with one exception; about twenty years ago one meeting was held in Louisville, Ky. It seems strange for this Society to meet anywhere else than in Cincinnati ; but we shall feel that it is perfectly safe in St. Louis ; for the brethren there know how to take care of such things. Indeed, we should have entire confidence that this Society would be honored and fostered wherever it might go. Its age and uniformly sterling course will command respect wherever it may be.

We trust there will be a large attendance of the membership, and may there be a large increase in this direction.

The subjects will be interesting and important.

### THE OHIO DENTAL COLLEGE ASSOCIATION.

The annual meeting of this Association will be held in the lecture room of the College on Tuesday, Feb'y. 24th, at 10 o'clock A. M., at which time and place it is very desirable that all members of the Association, so far as possible, be present, as matters of great interest to the Institution and the profession will be up for consideration and action. Then let all come, with the will and determination to do something for the good cause. This Institution has been a great agent for good in the profession; but it can be made still more effective, if it receives the aid and hearty co-operation of those who are interested in it.

### COMMENCEMENT.

The commencement exercises of the Ohio Dental College will be held in the lecture hall of the College on Tuesday evening, Feb. 24, at 7 1-2 o'clock, upon which occasion all the friends of the Institution and the public are cordially invited.

The exercises will be such as ordinarily pertain to such occasions.

### CONTENTS.

### COMMUNICATIONS.

### THE DENTAL REGISTER,

### OF THE WEST.

### A Monthly Magazine, Devoted Entirely to the Interest of the DENTAL PROFESSION.

**Terms Reduced to $2.50 per Year. Single Nos. 25 Cents.**

**Edited by PROF. J. TAFT,**

And Published by SPENCER & MOORE, of the Ohio Dental Depot

The volume commences in January and closes in December.

The Register being issued monthly is always fresh, therefore indispensable to the practitioner who desires to keep posted up with the new inventions and improvements which are daily developed. Neither expense nor effort will be spared to give value and variety to its contents. Articles will be illustrated with well executed engravings whenever they will add to the interest or assist in explanation.

Its extensive circulation and frequency of issue makes it one of the BEST DENTAL ADVERTISING MEDIUMS in the United States.

All subscriptions must begin with January or July.

**RATES OF ADVERTISING:**

| | 1 mo. | 3 mo. | 6 mo. | 1 year. | | 1 year. | |
|---|---|---|---|---|---|---|---|
| 1 page | $12 00 | $25 00 | $40 00 | $75 00 | Inserted pages must be plain | 1st page. | $50 00 |
| ½ page | 8 00 | 15 00 | 25 00 | 40 00 | paper & black | 2d page. | 37 50 |
| ¼ page | 5 00 | 10 00 | 15 00 | 25 00 | ink. | 3d page. | 33 33 |
| | | | | | | 4th page. | 25 00 |

Local or special notices, five lines or less, will be inserted for $3 each insertion; over five lines, 50 cents per line.

All advertising bills payable in advance, or at furthest, after the issue of the first number containing the advertisement. All transient advertisements to be paid for in advance.

☞CONTRIBUTIONS SOLICITED.

Exclusively Editorial Communications should be addressed to

**DR. J. TAFT, EDITOR.**

**The latest number of the Register may be ordered with or without other goods at any time, and all letters should be addressed to**

**SPENCER & MOORE. PUBLISHERS,**

Cincinnati, Ohio.

